# Abscisic acid biosynthesis, metabolism and signaling in ripening fruit

**DOI:** 10.3389/fpls.2023.1279031

**Published:** 2023-12-06

**Authors:** Wei Wu, Shi-feng Cao, Li-yu Shi, Wei Chen, Xue-ren Yin, Zhen-feng Yang

**Affiliations:** ^1^ College of Biological and Environmental Sciences, Zhejiang Wanli University, Ningbo, Zhejiang, China; ^2^ College of Food Science and Engineering, Ocean University of China, Qingdao, Shandong, China; ^3^ Zhejiang Provincial Key Laboratory of Integrative Biology of Horticultural Plants, Department of Horticulture, Zhejiang University, Hangzhou, Zhejiang, China

**Keywords:** fruit ripening, abscisic acid, phytohormone interaction, transcriptional regulation, ethylene

## Abstract

Fruits are highly recommended nowadays in human diets because they are rich in vitamins, minerals, fibers and other necessary nutrients. The final stage of fruit production, known as ripening, plays a crucial role in determining the fruit’s quality and commercial value. This is a complex physiological process, which involves many phytohormones and regulatory factors. Among the phytohormones involved in fruit ripening, abscisic acid (ABA) holds significant importance. ABA levels generally increase during the ripening process in most fruits, and applying ABA externally can enhance fruit flavor, hasten softening, and promote color development through complex signal regulation. Therefore, gaining a deeper understanding of ABA’s mechanisms in fruit ripening is valuable for regulating various fruit characteristics, making them more suitable for consumption or storage. This, in turn, can generate greater economic benefits and reduce postharvest losses. This article provides an overview of the relationship between ABA and fruit ripening. It summarizes the effects of ABA on ripening related traits, covering the biochemical aspects and the underlying molecular mechanisms. Additionally, the article discusses the interactions of ABA with other phytohormones during fruit ripening, especially ethylene, and provides perspectives for future exploration in this field.

## 1 Introduction

In modern human diets, fruit is highly recommended due to its abundant vitamins, minerals, fibers, and essential nutrients. The final stage of fruit production, known as ripening, has a direct impact on the overall quality and commercial value of fruit (Giovannoni et al., 2017). This intricate process involves significant transformations in texture, color, flavor, and other attributes (Li et al., 2021; Li et al., 2022). Gaining an understanding of the mechanisms underlying fruit ripening is instrumental in regulating specific fruit characteristics, making them more suitable for consumption or storage. This, in turn, can lead to greater economic benefits and reduced postharvest losses.

Phytohormones serve as signaling molecules produced by plants and play crucial roles in fruit development and ripening processes, even at low concentrations (Tucker et al., 2017; Li et al., 2021). Among these hormones, abscisic acid (ABA) stands out with its sesquiterpenoid structure, and its discovery dates back to the 1960s (Addicott and Lyon, 1969). ABA has been found to be widely present in different plant tissues and organs, where it regulates processes such as seed dormancy (Ali et al., 2021), leaf senescence (An et al., 2021b), stomatal movement (Hsu et al., 2021), and plant stress responses (Lim et al., 2022). Notably, ABA has also been identified as a crucial factor during the ripening of numerous fruits, significantly accelerating processes like color development, softening, and sugar accumulation (Oh et al., 2018; Liang et al., 2020). Fleshy fruits can generally be classified as climacteric or non-climacteric fruits based on whether there is a distinct surge in respiration during the ripening process. Climacteric fruits undergo a sharp increase in respiration as they ripen, such as tomato, kiwifruit, persimmon, and banana. On the other hand, non-climacteric fruits do not exhibit a significant burst of respiration during ripening, such as citrus, strawberry, and grape (Li et al., 2020). Early researches on the regulation of ABA primarily focused on non-climacteric fruits, while studies on climacteric fruits mainly revolved around ethylene. However, in recent years, there has been a growing interest in exploring the role of ABA in climacteric fruits. Understanding how ABA controls fruit ripening and its interaction with ethylene and other hormones has become a topic of significant research interest.

In this article, we provide an overview of the relationship between ABA and fruit ripening. We summarize the impact of ABA on both climacteric and non-climacteric fruits, with a particular focus on the underlying molecular mechanisms. Additionally, the interactions of ABA with other phytohormones, especially ethylene, during fruit ripening are discussed. These recent perspectives and systematic summaries may enrich our understanding of the relationship between ABA and fruit ripening, and provide a theoretical basis for developing more accurate and efficient technologies for fruit quality regulation.

## 2 ABA biosynthesis and metabolism during fruit ripening

The levels of ABA tend to be closely related to fruit ripening in both climacteric and non-climacteric fruits. Figueroa et al. (2021) detected the free ABA contents in six analyzed strawberry cultivars and found the contents all increased during fruits ripening. In sweet orange, ABA levels peak in the colored stage in both Navelate and Pinalate cultivars (Romero and Lafuente, 2020). During the ripening of mango fruit, the ABA content gradually increases with changes in total soluble solids (TSS), firmness and color (Wu et al., 2022a). In tomato fruit, ABA content also increases and reaeches its peak at breaker stage (Wang et al., 2021).

**Figure 1:**
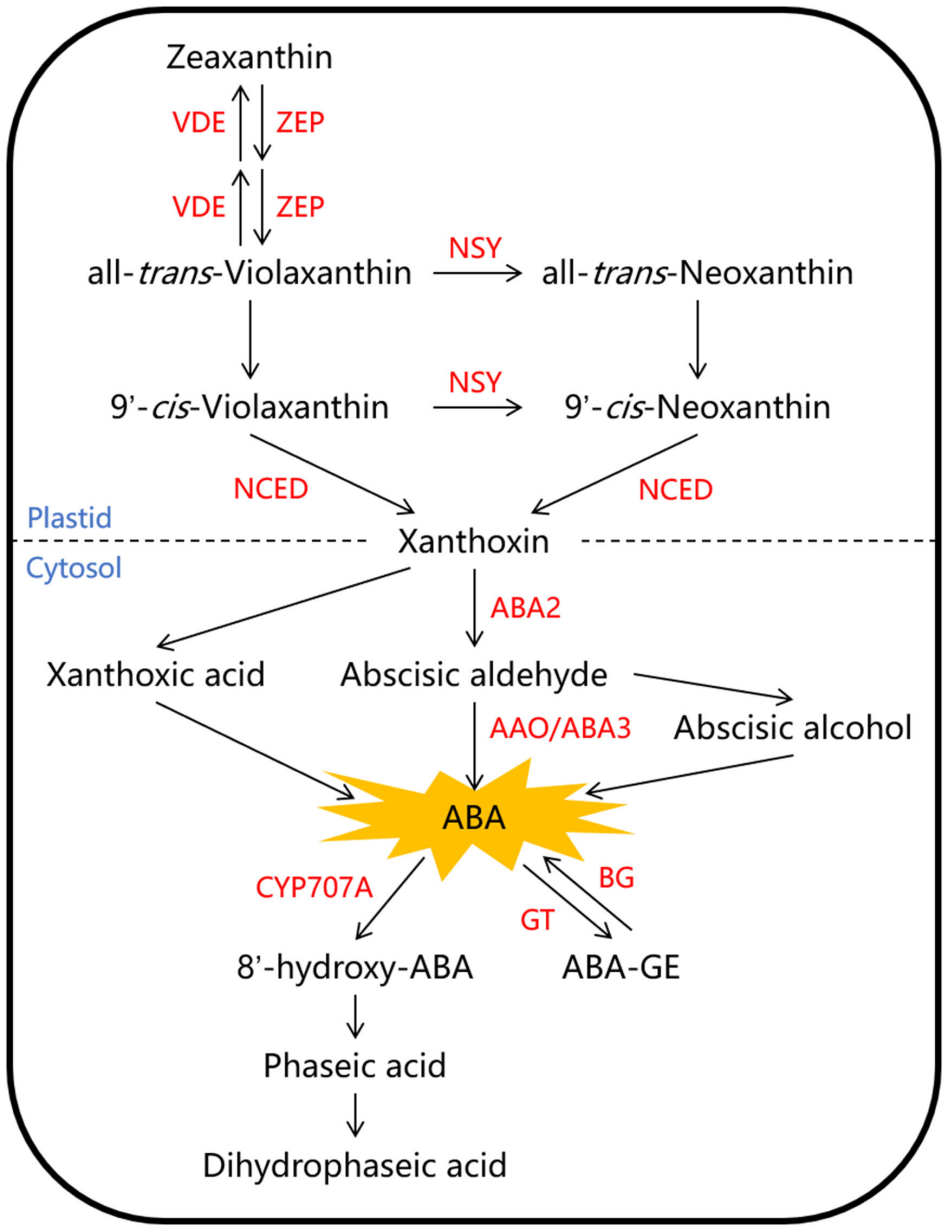
The biosynthesis and metabolism pathways of abscisic acid (ABA) in higher plant. The C_40_ zeaxanthin is first converted into all-*trans*-violaxanthin under the catalysis of zeaxanthin epoxidase (ZEP). Then, all-*trans*-violaxanthin can be converted to 9’-*cis*-violaxanthin or 9’-*cis*-neoxanthin in two pathways under the catalysis of neoxanthin synthase (NSY) and the unknown isomerase. The enzyme 9-*cis*-cyclocarotenoid dioxygenase (NCED) cleaves both 9’-*cis*-neoxanthin and 9’-*cis*-violaxanthin into xanthoxin. Eventually, xanthoxin is mainly converted into abscisic aldehyde and further forms ABA, which are catalyzed by a short-chain alcohol dehydrogenase (ABA2) and abscisic aldehyde oxidase (AAO/ABA3) respectively. ABA metabolism involves two main pathways: hydroxylation and glycosylation, which are catalyzed by the cytochrome P450 monooxygenase (CYP707A) and ABA glucosyltransferase (GT) respectively. Red letters represent enzymes, and blue letters represent cellular components divided by dashed line. ABA-GE, ABA-glucose ester; BG, β-glucosidase; VDE, violaxanthin de-epoxidase.

The ABA content in fruit is mainly controlled by its biosynthesis and metabolism. The primary pathway for ABA biosynthesis in higher plants is believed to be the C_40_ carotenoid indirect pathway, and the associated enzymes involved in this pathway have been largely characterized and well summarized in Arabidopsis (**Figure 1**). The process begins with the synthesis of a C_40_ carotenoid precursor in the plastids, and is followed by a series of isomerization and cleavage reactions leading to the formation of C_15_ xanthoxin. The C_40_ zeaxanthin is first converted into all-*trans*-violaxanthin under the catalysis of zeaxanthin epoxidase (ZEP). Then, all-*trans*-violaxanthin can be directly catalyzed by an unknown isomerase to form 9’-*cis*-violaxanthin, or it can be converted to all-*trans*-neoxanthin first under the catalysis of neoxanthin synthase (NSY), and then to 9’-*cis*-neoxanthin under the catalysis of an unknown isomerase. The enzyme 9-*cis*-cyclocarotenoid dioxygenase (NCED) cleaves both 9’-*cis*-neoxanthin and 9’-*cis*-violaxanthin into xanthoxin, which is considered to be the rate-limiting step in ABA biosynthesis. Eventually, xanthoxin is mainly converted into abscisic aldehyde and further forms ABA, which are catalyzed by a short-chain alcohol dehydrogenase (ABA2) and abscisic aldehyde oxidase (AAO/ABA3) respectively in the cytosol (Nambara and Marion-Poll, 2005; Li et al., 2022). ABA metabolism involves two main pathways: hydroxylation and glycosylation (**Figure 1**). The conversion of ABA to phaseic acid is a crucial step in ABA metabolism, primarily facilitated by the cytochrome P450 monooxygenase (CYP707A) at the 8’-methyl position (Liao et al., 2018). Additionally, active ABA can become inactive through glycosylation, which is catalyzed by ABA glucosyltransferase (GT) and forms ABA-glucose ester (ABA-GE); The reverse reaction can also occur, converting ABA-GE back to ABA, and this process can be catalyzed by β-glucosidase (BG) (Sun et al., 2017; Liang et al., 2020).

Several studies have cloned the *NCED* family genes and observed changes in their expression that correlated with ABA content during the ripening of various fruits, such as strawberry, tomato and persimmon (Jia et al., 2011; Liang et al., 2020; Zuo et al., 2020). For example, the expression of *NCED5* is upregulated during the transformation of mature green to red ripe stages of tomato fruit (Zuo et al., 2020). The expression of *PpeNCED2* increases during fruit ripening in several peach cultivars (Wang et al., 2019a). The ABA level is found to be down-regulated after the silencing of *FaNCED1* in strawberry, and this phenotype can be rescued by ABA treatment, verifying that *FaNCED1* controls strawberry ripening via influencing ABA biosynthesis (Jia et al., 2011). Apart from NCEDs, other enzymes involved in ABA biosynthesis have also been reported to participate in fruit ripening. For instance, the expression of zeaxanthin epoxidase gene *MiZEP* increases at the late ripening stage of mango fruit (Wu et al., 2022a). And the expression of *VmNSY* increases significantly in berry tissue with the accumulation of ABA (Karppinen et al., 2013). However, compared to *NCEDs*, there are relatively fewer studies investigating other genes in the ABA biosynthesis pathway during fruit ripening. Additionally, the expression of β-glucosidase gene *DkBG1* is observed to increase during fruit ripening, which promotes the release of free ABA, and ectopic overexpression of *DkBG1* in tomato results in significantly elevated ABA levels (Liang et al., 2020). The transcript level of *FveCYP707A4a*, which is involved in the hydrolysis of ABA, decreases dramatically at the ripening initiation phase (Liao et al., 2018). The expression trends of these ABA biosynthetic and metabolic genes provide evidence for the vital role of ABA in fruit ripening. However, the expression of some ABA-metabolizing genes, such as *SlUGT75C1*, *SlUGT73C4*, and *SlUGT76E1*, involved in the inactivation of ABA, is found to be highly upregulated during fruit ripening, which may be a feedback loop employed by plants to control the levels of ABA (Sun et al., 2017).

## 3 Exogenous ABA treatment regulates fruit ripening

In addition to the correlation between ABA content and fruit ripening, the application of exogenous ABA has also assisted in demonstrating its importance in fruit ripening traits. Color is an intuitive character reflecting the maturity of fruit. The color of fruit comes from changes in pigment composition, including chlorophylls that appear green, carotenoids that appear yellow to red, and anthocyanins that appear red to purple. Compared with control fruit, the flesh color of mango treated with ABA is yellower and the expression of carotenoids biosynthesis gene *MiPSY* increases, whereas the ABA inhibitor fluridone inhibits carotenoids accumulation (Wu et al., 2022a). Similarly, exogenous ABA treatment accelerates carotenoids accumulation and chlorophylls degradation in cherry tomato (Wu et al., 2018). The biosynthesis of anthocyanins can also be accelerated by ABA treatment in blueberry, fig, litchi and strawberry fruit (Hu et al., 2018; Oh et al., 2018; Li et al., 2019; Lama et al., 2020). The application of ABA can improve grape fruit skin color characteristics by inducing anthocyanins accumulation (Peppi et al., 2006). The expression of *C4H*, *CHI*, *CHS*, *DFR*, *GTs*, *LDOX*, and *PAL*, genes involved in anthocyanins and flavonoids biosynthesis, can be upregulated by exogenous ABA treatment (Hu et al., 2018). The contents of cyanidin 3-O-glucoside and cyanidin 3-O-rutinoside, the major compositions of anthocyanins in fig fruit, increase under ABA treatment, while the expression of anthocyanins biosynthesis genes such as *FcANS*, *FcCHI*, *FcCHS2*, *FcDFR*, *FcF3H*, *FcPAL* and *FcUFGT*, is downregulated by ABA inhibitors nordihydroguaiaretic acid (NDGA) and fluridone (Lama et al., 2020). ABA was found to mainly accelerate the accumulation of delphinidin, malvidin and petunidin glycosides in blueberry (Oh et al., 2018).

Flavor is also a key attribute to fruit ripening quality, composed of taste and aroma, involving components such as sugars, acids and aromatic volatiles. Researches show that ABA treatment markedly promote starch degradation and increase the contents of soluble sugars (glucose and sucrose) in apple fruit. The expression of *MdTMT1* and *MdSUT2* genes, responsible for glucose and sucrose transport respectively, and the amylase coding genes *MdAMY1/3* and *MdBAM1/3* is induced in response to ABA (Ma et al., 2017). The TSS content is found to increase after ABA treatment in mango and it is inhibited by fluridone (Wu et al., 2022a). ABA treatment maintains soluble sugars content of peach fruit stored cold, via increasing the activities of sucrose phosphate synthase and sucrose synthase (Zhao et al., 2022). During cherry tomato ripening, the aromatic volatiles production is improved by ABA, including fatty acid-derived, amino acid-derived and branched-chain volatiles (Wu et al., 2018). NDGA treatment downregulates the expression of genes such as *VvADH*, *VvGPPS*, *VvLOX* and *VvTPS* in the aroma metabolism to affect berry fruit aroma volatile synthesis (Lin et al., 2018).

The speed-up of fruit ripening by ABA treatment is also reflected in softening (Wu et al., 2022a). The application of ABA can increase grape fruit softening (Peppi et al., 2006). ABA treatment significantly decreases sweet cherry fruit firmness, and NDGA treatment inhibits fruit softening, via regulating the expression of cell wall modifying genes *PavPL18*, *PavPME44* and *PavXTH26/31* (Zhai et al., 2022). Other ripening traits, such as ethylene and ascorbic acid contents, can also be increased by ABA treatment (Xu et al., 2022; Wu et al., 2022b). The prolonged water loss in the ABA-deficient mutant orange ‘Pinalate’ can be relieved by ABA application (Romero et al., 2013). Exogenous ABA can activate the ascorbate-glutathione cycle to alleviate the chilling injury of peach fruit (Tang et al., 2022). However, there are few studies in this area, and the effects of ABA on other secondary metabolites or nutrients during fruit ripening are worth further investigation. Overall, ABA shows a promoting effect on fruit ripening, primarily controlling fruit color, flavor and texture by regulating the expression levels of related metabolic pathway genes, both in climacteric and non-climacteric fruits (**Table 1**).

**Table 1:** Effects of ABA on the ripening of some fruits.

Fruits	Treatments		Effects				References
	Concentration	Method	Color	Flavor	Texture	Other traits	
Apple	0.02 mM	Sprayed for 4 day	–	√	–	–	Ma et al., 2017
Blueberry	1 g/L	Dipped for 1 min	√	–	√	–	Oh et al., 2018
Cherry tomato	1 mM	Infiltrated under 60 kPa vacuum for 3 min	√	√	√	Promote ethylene production	Wu et al., 2018
Citrus	1 mM	Dipped for 2 min	–	–	–	Reduce weight loss	Romero et al., 2013
Fig	1.89 mM	Injected with a syringe	√	–	–	–	Lama et al., 2020
Grape	300 mg/L	Sprayed	√	–	–	–	Peppi et al., 2006
Litchi	25 mg/L	Sprayed	√	–	–	–	Hu et al., 2018
Mango	1 mM	Dipped for 10 min	√	√	√	–	Wu et al., 2022a
Melon	0.05 mM	Injected with a syringe	–	√	√	Promote ethylene production	Sun et al., 2013
Peach	0.1 mM	Dipped for 10 min	–	√	–	Reduce flesh browning	Zhao et al., 2022
Persimmon	300 mg/L	Sprayed every half day for three times	√	–	√	Promote ethylene production	Wu et al., 2022b
Strawberry	0.1 mM	Injected with a syringe	√	–	–	–	Li et al., 2019
Sweet cherry	0.2 mM	Sprayed every 1-2 days for four times	–	–	√	–	Zhai et al., 2022
Tomato	0.1 mM	Sprayed every other day for 10 days	–	–	–	Accumulate ascorbic acid	Xu et al., 2022

The " √ " means that this trait is reported to be affected by ABA treatment. The “-” indicates that it is not mentioned in the paper.

## 4 ABA signaling during fruit ripening

**Figure 2:**
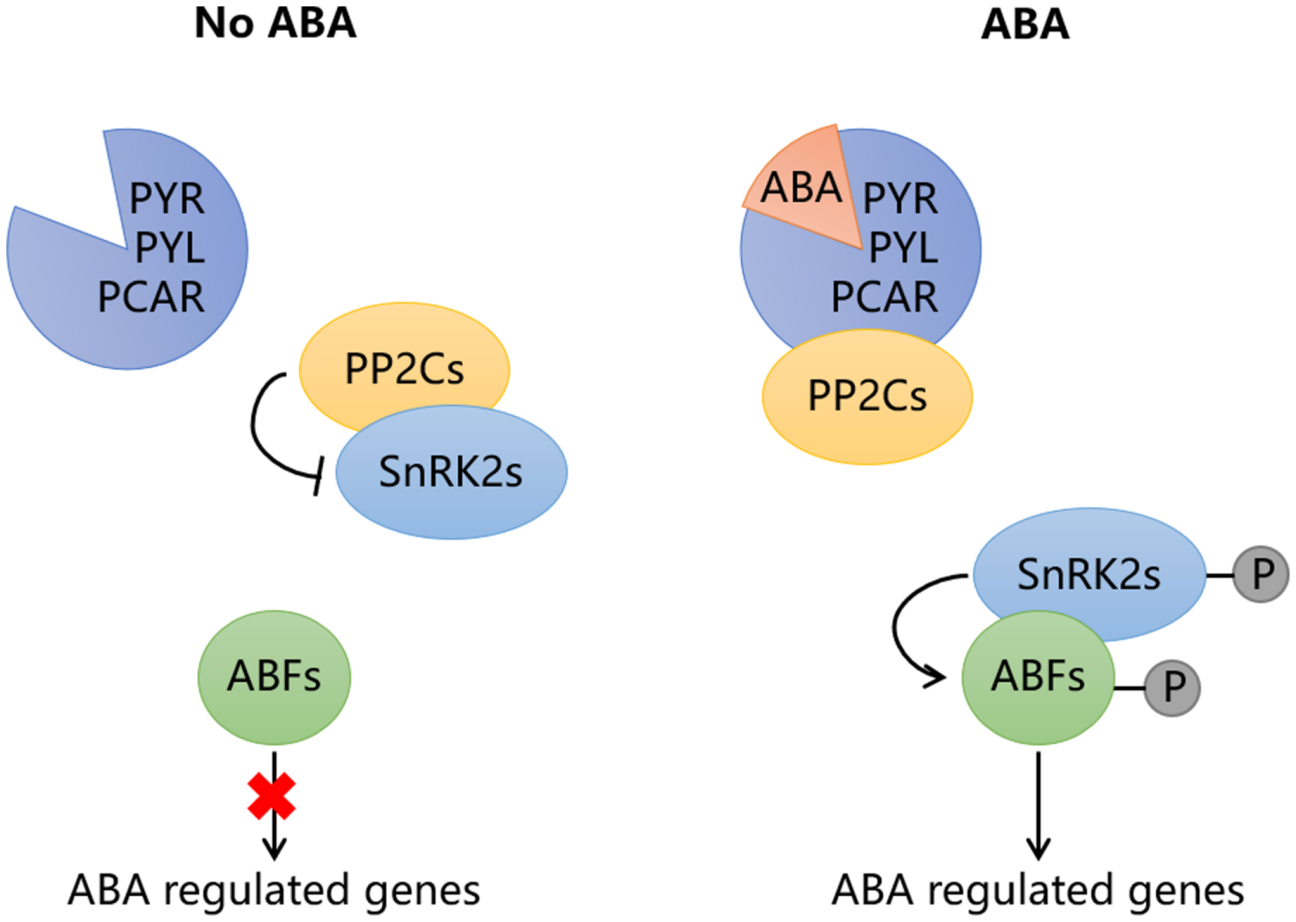
The signal transduction pathway of abscisic acid (ABA). In the absence of ABA, the type 2C protein phosphatases (PP2Cs) interact with the SNF1-related kinases (SnRK2s) to block ABA signaling. When ABA exists, the ABA receptor PYR/PYL/PCAR can form complex with PP2Cs after binding to ABA, so that SnRK2s are released. The released SnRK2s are phosphorylated by themselves or other kinases, then activate the ABA-responsive element binding factors (ABFs), which are involved in the regulation of target downstream genes. The red cross means this path is blocked.

**Figure 3:**
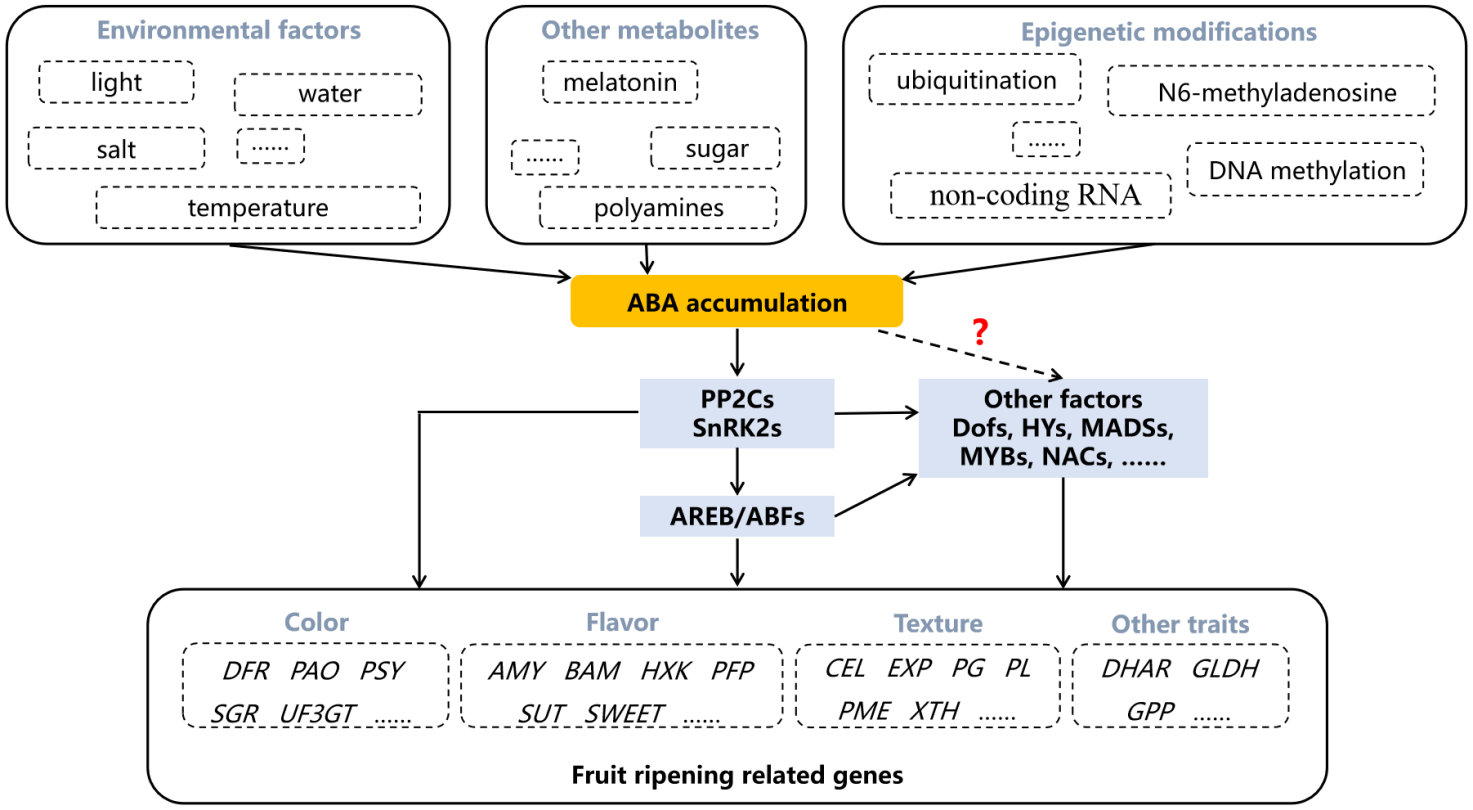
The regulation of abscisic acid (ABA) in fruit ripening. Dotted arrow and question mark indicate the regulatory pathway that is not yet clear. AMY, amylase; AREB/ABFs, ABA responsive element binding factors; BAM, beta- amylase; CEL, cellulase; DFR, dihydroflavonol 4-reductase; DHAR, dehydroascorbate reductase; EXP, expansin; GLDH, L-galactono-1,4-lactone dehydrogenase; GPP, L-galactose-1-phosphate phosphatase; HXK, hexokinase; PAO, pheophorbide a monooxygenase; PFP, fructose-6-phospho1-phosphotransferase; PG, polygalacturonase; PL, pectinlyase; PME, pectin methylesterase; PP2Cs, type 2C protein phosphatases; PSY, phytoene synthase; SnRK2s, SNF1-related kinases; SGR, staygreen; SUT, sucrose transporter; UF3GT, UDP-glucose flavonoid 3-o-glucosyltransferase; XTH, xyloglucan endotransglycosylase/hydrolase.

The signal transduction of ABA mainly depends on four core components, including ABA receptors PYR/PYL/PCAR, type 2C protein phosphatases (PP2Cs), the subfamily 2 of the SNF1-related kinases (SnRK2s) and ABA responsive element binding proteins (AREB/ABFs) (**Figure 2**; Chen et al., 2020). When ABA binds to PYR/PYL/PCAR protein, the protein structure changes, enabling it to form a complex with PP2C, which disrupts the interaction between PP2C and SnRK2. As a result, the released SnRK2 is phosphorylated either by itself or by other kinases, subsequently activating the AREB/ABFs. In the absence of ABA, PP2C interact with SnRK2 to block ABA signaling. This PYR/PYL/PCAR-PP2Cs-SnRK2s-AREB/ABFs signaling pathway is conserved both in climacteric and non-climacteric fruits (**Figure 3**). In tomato, the expression of ABA receptor coding gene *SlPYL9* continually increases after the onset of breaker stage with the concomitant ripening phenotype. Both overexpression and repression of *SlPYL9* alter the ripening process (Kai et al., 2019). Likewise, the expression of *SlSnRK2C* is also reported to increase during tomato fruit ripening (Sun et al., 2011). Contrarily, the transcription of *FaABI1* encoding PP2Cs decreases rapidly during strawberry fruit ripening; The silencing of this gene affects the transcripts of many ABA-responsive genes, and accelerates ripening process, which confirmed its negative role in ABA signaling (Jia et al., 2013). Recently, ClSnRK2.3 has been found to directly phosphorylate the pyrophosphate-dependent phosphofructokinase ClPFP1, which is involved in sugar metabolism pathway, to accelerate its degradation and this process finally results in low sucrose content in watermelon fruit (Wang et al., 2023), indicating that ABA signaling may be transmitted without AREB/ABFs and the intermediate components such as PP2Cs and SnRK2 may directly regulate target genes related to fruit ripening. However, extensive studies have revealed that ABA signals are mainly transmitted to downstream target genes through AREB/ABFs. MdAREB2 promotes soluble sugar accumulation by regulating the expression of sugar transporter gene *MdSUT2*, and amylase genes *MdAMY1/2* and *MdBAM1/3* (Ma et al., 2017). In litchi fruit, LcABF1/2 are involved in coloration by targeting the promoter region of *PAO* and *SGR* genes that control chlorophylls degradation (Hu et al., 2019). Furthermore, LcABF2/3 can bind to the promoter of *LcMYB1*, which plays a role in regulating anthocyanins biosynthesis (Hu et al., 2019). The abscisic acid-insensitive5 ABI5-like protein, which belongs to AREB/ABFs, can bind and regulate the starch and cell wall metabolism-related genes, such as *BAM8* and *PL8* (Song et al., 2022). In addition to directly acting on ripening-related structural genes, AREB/ABFs can also affect fruit ripening by regulating other TFs. For example, SlAREB1 is a direct activator of *NOR*, which is a bZIP TF involved in ethylene synthesis, color formation and cell wall metabolism in tomato (Bastias et al., 2014; Gao et al., 2018; Mou et al., 2018). Moreover, MdABI5 is reported to directly activate *MdbHLH3* to target anthocyanin biosynthesis genes *MdDFR* and *MdUF3GT* (An et al., 2021a).

Besides, some other components involved in ABA signaling have also been reported to participate in fruit ripening, especially in non-climacteric fruits. Like another ABA receptor protein CHLH/ABAR. The silencing of *FaCHLH/ABAR* in strawberry alters ABA level and influences fruit coloring as well as sugar accumulation (Jia et al., 2011). What’s more, the kinase FaRIPK1 can interact with FaABAR to regulate the ripening process involved with softening, sugar accumulation and coloring, and a novel signaling pathway FaABAR-FaRIPK1-FaABI4 in strawberry is proposed (Hou et al., 2018). However, these studies remain inadequate in climacteric fruits, and the presence of a similar pathway is yet to be investigated. Additionally, other regulators involved in ABA signaling have been implicated in fruit ripening. The ABA-suppressed FaMADS1a plays a negative role in strawberry fruit ripening, which represses the expression of *FaANS*, *FaCHS*, *FaDFR* and *FaPAL6* to inhibit anthocyanins accumulation (Lu et al., 2018). ABA-responsive MiHY5 can directly activate the expression of sugar accumulation-related genes *MiSWEETs*, *MiHXK1*, *MiBAM9*, and carotenoids biosynthesis gene *MiPSY* (Wu et al., 2022a). Under the influence of ABA-induced transcription factor PavDof6, the expression of genes associated with cell wall degradation, named *PavPME44*, *PavPL18*, *PavXTH31*, and *PavXTH26*, are up-regulated, however, the inhibition of PavDof2/15 by ABA decreases the expression of these genes (Zhai et al., 2022). Analogously, ABA-induced PavNAC56 directly activate the expression of cell wall-related genes *PavPG2*, *PavEXPA4*, *PavPL18* and *PavCEL8* (Qi et al., 2022). Ethylene biosynthesis genes *SlACS2/4*, color-related genes *SlGgpps2* and *SlSGR1*, and cell-wall-related genes *SlPG2a*, *SlPL*, *SlCEL2* and *SlEXP1*, are all direct targets of ABA-induced NAC TF NOR-like1 (Gao et al., 2018). Moreover, ABA can induce protein kinase SlMAPK8 to phosphorylate SlMYB11, which then activates the expression of *GPP*, *GLDH* and *DHAR* to accumulate ascorbic acid (Xu et al., 2022). However, the response of these regulators to ABA and the coordination mechanisms between various signaling pathways require further exploration.

Overall, ABA affects fruit ripening through multiple pathways. It can participate in the regulation of fruit ripening not only through conservative signaling pathway, but also by influencing other signaling components that respond to ABA treatment. On the one hand, these signaling components can directly regulate the expression of metabolic pathway genes related to fruit ripening, or related protein activity; On the other hand, they can also regulate the regulatory factors of these metabolic pathway genes to affect fruit ripening.

## 5 Crosstalk between ABA and other phytohormones to affect fruit ripening

**Figure 4:**
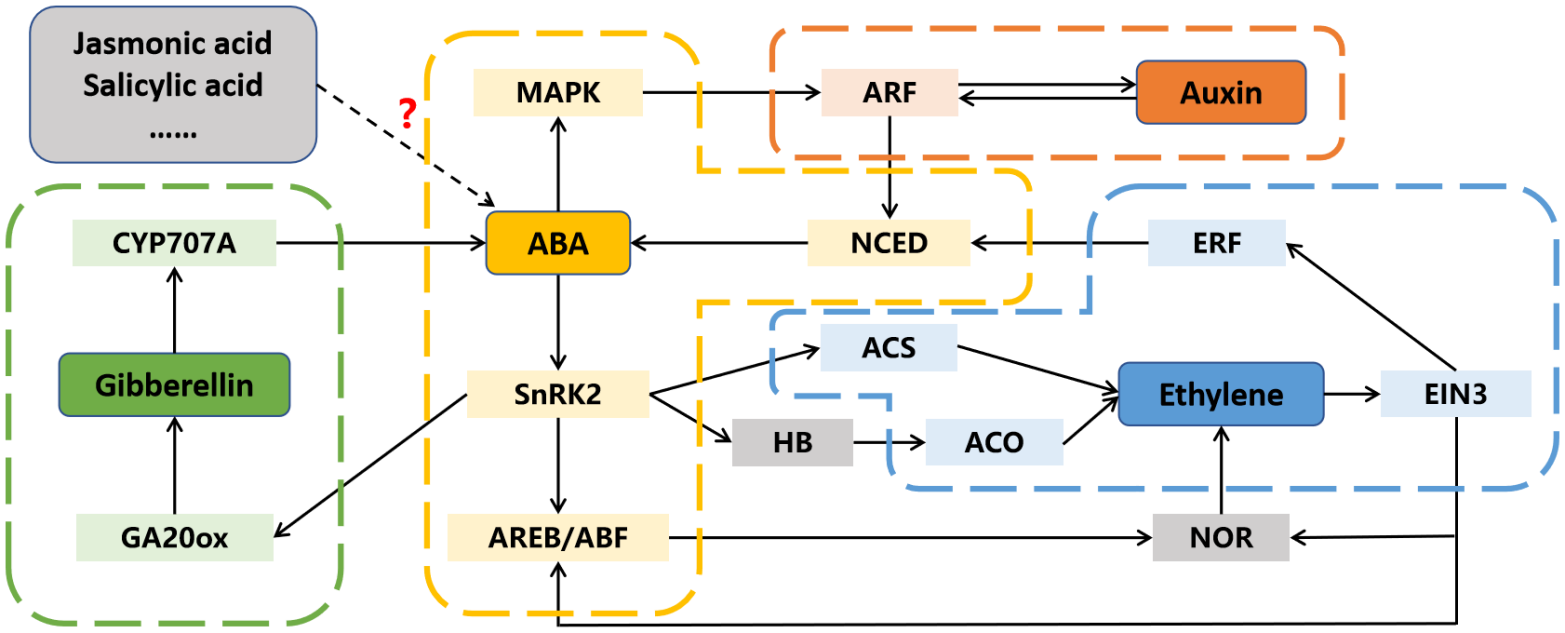
The interactions between abscisic acid (ABA) and other phytohormones in fruit ripening. Dotted arrow and question mark indicate the interaction is not yet clear. ACO, 1-aminocyclopropyl-carboxylic acid oxidase; ACS, 1-aminocyclopropyl-carboxylic acid synthase; AREB/ABF, ABA responsive element binding factor; ARF, auxin response factor; CYP707A, cytochrome P450 monooxygenase; EIN3, ethylene insensitive 3; ERF, ethylene response factor; GA20ox, GA 20-oxidase; HB, homeobox transcription factor; MAPK, mitogen activated protein kinase; NCED, 9-cis-cyclocarotenoid dioxygenase; NOR, non-ripening; SnRK2s, SNF1-related kinases.

After decades of in-depth researches on a single hormone, people have gradually realized the importance of interactions between different hormones (Kai et al., 2019; Aerts et al., 2021). In addition to ABA, several other phytohormones participate in fruit ripening, and there is evidence to suggest that ABA may interact with them to regulate this process (**Figure 4**; Li et al., 2017; Deng et al., 2018; Garrido-Bigotes et al., 2018).

### 5.1 Interactions between ABA and ethylene

Ethylene is recognized as the primary phytohormone involved in fruit ripening, and its regulatory mechanism has been extensively studied and reviewed (Li et al., 2020). However, the initial trigger for ethylene biosynthesis at the onset of fruit ripening remains to be elucidated. In many fruits, the peak of ABA content precedes that of ethylene during the ripening process, suggesting that ABA may regulate ripening by inducing ethylene release (Wang et al., 2021; Wu et al., 2022b). Numerous pieces of evidence support the idea that ABA can regulate ethylene biosynthesis in various fruits. For instance, the silencing of *ZDS*, a gene involved in ABA biosynthesis, resultes in a decrease in ABA content and prolongs tomato fruit ethylene production (McQuinn et al., 2020). In contrast, the increase in ABA content led by the suppression of *SlUGT75C1*, also induces ethylene release (Sun et al., 2017). Similarly, overexpression of *DkBG1*, which hydrolyzes ABA-glucose ester to release ABA, accelerates ethylene release by increasing ABA level (Liang et al., 2020). Moreover, the regulation of ABA signaling-related gene expression also affects ethylene biosynthesis. Both overexpression and repression of *SlPYL9* alter the transcription of ethylene-related genes, including *SlACS2*, *SlACS4*, *SlACO1*, *SlCTR1*, *SlETR3*, and *SlERF2* (Kai et al., 2019). SlAREB1 has been reported to directly activate the expression of *NOR* by binding to its promoter, thereby accelerates ethylene release and tomato fruit ripening (Mou et al., 2018).

While ABA’s involvement in ethylene biosynthesis during fruit ripening is well-established, the influence of ethylene on ABA biosynthesis in fruit has also been reported. Recent researches demonstrate that 1-methylcyclopropene (an ethylene inhibitor) treatment reduces ABA content in peach fruit, and the transcription factors PpERF3 and PpeERF2 are found to directly activate or suppress the expression of ABA biosynthetic genes *NCEDs*, indicating that ethylene can also influence ABA biosynthesis in fruit (Wang et al., 2019a; Wang et al., 2019b). Moreover, ethylene signaling component EBF1 can physically interact with ABI5-like to enhance its regulation on starch and cell wall degradation related genes (Song et al., 2022). Another component EIN3 can inhibit ABI4 to regulate ABA biosynthesis via repressing the expression of *VTC2* (Yu et al., 2019).

### 5.2 Interactions between ABA and auxin

A substantial decrease in auxin content, along with an increase in ABA level, is observed from fruit set to ripening, and exogenous ABA treatment decreases auxin content, suggesting a potential antagonistic relationship between auxin and ABA during fruit ripening (Jia et al., 2017). ABA is reported to repress the expression of auxin response factor *PavARF8*, and PavARF8 directly bind to the promoter of ABA biosynthesis gene *PavNCED1*, forming a regulation loop (Zhai et al., 2022). Moreover, ABA-induced protein kinase SlMAPK8 can phosphorylate SlARF4 and SlMYB11 to respectively inhibit and activate their transcription activity. Meanwhile, auxin-induced SlARF4 is the inhibitor of SlMYB11, modulating its regulation of ascorbic acid (Xu et al., 2022). Thus, both ABA and auxin are involved in tomato fruit ascorbic acid accumulation by the SlMAPK8-SlARF4-SlMYB11 pathway.

### 5.3 Interactions between ABA and other phytohormones

Crosstalk between ABA and other phytohormones during fruit ripening has also been documented. A significant decrease in jasmonic acid (JA) content, along with an increase in ABA levels, is observed from the flowering to ripening stages of strawberry fruit (Garrido-Bigotes et al., 2018). However, exogenous methyl-JA treatment triggers anthocyanins and sucrose accumulation and a reduction in ABA, and overexpression of JA biosynthesis genes *FaAOC* and *FaAOS* promotes strawberry fruit ripening (Garrido-Bigotes et al., 2018; Han et al., 2019). Additionally, the expression of *ASR*, a TF involved in fruit ripening, is influenced not only by ABA but also by auxin and JA, showing that these phytohormones are correlated during fruit ripening (Jia et al., 2016). Moreover, gibberellic acid (GA) can activate the removal of ABA through FveCYP707A4a-induced metabolism, indicating that down-regulation of GA may result in ABA accumulation and promote fruit ripening (Liao et al., 2018). However, ABA signaling component ClSnRK2.3 phosphorylates GA biosynthesis enzyme ClGA20ox to accelerate its degradation, which results in low GA level and negatively regulates watermelon fruit ripening (Wang et al., 2023).

The above findings indicate that ABA can not only directly affect fruit ripening, but also participate in this process through hormone interaction such as inducing ethylene synthesis, inhibiting auxin signal transduction, and GA accumulation.

## 6 Regulation of ABA affects fruit ripening

With the determination of the role of ABA in regulating fruit ripening, some environmental factors, other metabolites and epigenetic modifications are found to participate in regulating fruit ripening by affecting ABA content. For example, mild drought and salt stresses result in anthocyanins accumulation accompanied by increased ABA content (Perin et al., 2019). Both red and blue light up-regulate *NCED* genes, resulting in ABA-regulated coloration (Samkumar et al., 2021). The increase of ABA content in berry skins is reduced by high temperature treatment, resulting in the greatly inhibition of anthocyanins accumulation (Ryu et al., 2020). In addition to environmental factors, other metabolites can also affect ABA biosynthesis. Exogenous polyamines spermine and spermidine promote strawberry fruit coloration, which is accompanied by changes in ABA content (Guo et al., 2018). The endogenous ABA level and the expression of ABA-related genes, such as *NCED1/2*, are significantly enhanced by sucrose treatment (Luo et al., 2020). Melatonin treatment increases ABA level and promotes grape berry ripening (Xu et al., 2018).

As a hot research topic in recent years, epigenetic modification is also involved in the regulation of ABA in fruit. The E3 ligase VlPUB38 interacts with abscisic-aldehyde oxidase VlAAO to mediate its degradation, negatively regulating fruit ripening (Yu et al., 2021). The differentially expressed non-coding RNA MSTRG.181568.2 during tomato fruit ripening is reported to potentially target *NCED* gene (Zuo et al., 2020). Ripening-related DNA demethylation is accompanied by ABA biosynthesis, and RNA methylation enhances the stability of NCED5 (Cheng et al., 2018; Zhou et al., 2021). These results further demonstrate the essential role of ABA in fruit ripening regulation, and make it possible to regulate fruit ripening traits by altering ABA synthesis and metabolism through genetic engineering techniques or appropriate exogenous treatments.

## 7 Conclusions and future perspective

Extensive researches have been conducted to investigate the role of ABA in regulating seed dormancy, plant growth, development, and its involvement in fruit ripening. ABA accumulation is considered a crucial switch that triggers the ripening process in many fruits, and the application of exogenous ABA has shown to enhance fruit flavor, accelerate softening, coloring, and other ripening-related changes by affecting the expression of related metabolic genes or the activity of related enzymes. ABA can regulate them through its signaling pathway directly, or through the regulatory factors acting on them, or by interaction with other phytohormones. The application of genetic engineering technologies, including gene silencing and overexpression, confirms the important roles of key genes involved in ABA biosynthesis and metabolism in fruit ripening regulation. In addition, various environmental factors and epigenetic modifications are reported to regulate fruit ripening via ABA.

In recent years, there has been significant progress in identifying various components involved in ABA signal transduction in fruits. Nonetheless, the roles of numerous genes related to ABA signaling remain contentious and require further analysis. It is essential to explore their relationship with the proposed signaling pathway and understand the circumstances under which these different pathways function. Additionally, considering the regulatory roles of other phytohormones like salicylic acid (Wang et al., 2022) and brassinosteroid (He et al., 2021) in fruit ripening, it becomes necessary to conduct comprehensive studies on their interactions with ABA. Moreover, investigating and comprehending the variations that exist among different fruit types are crucial. These investigations will significantly contribute to achieving precise control of specific fruit characteristics at the molecular level, thereby better meet the diverse needs of consumers.

## Author contributions

WW: Investigation, Writing – original draft. S-FC: Writing – review & editing. L-YS: Project administration, Writing – review & editing. WC: Project administration, Writing – review & editing. X-RY: Conceptualization, Writing – review & editing. Z-FY: Writing – review & editing.
